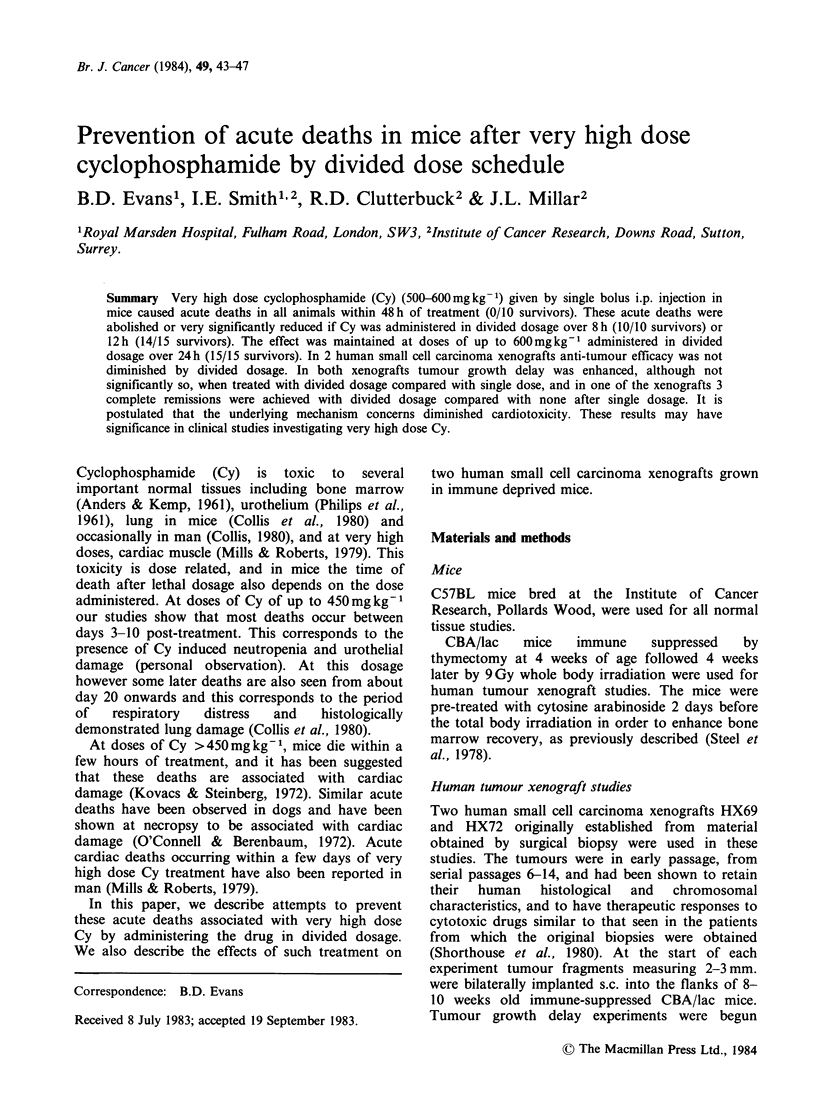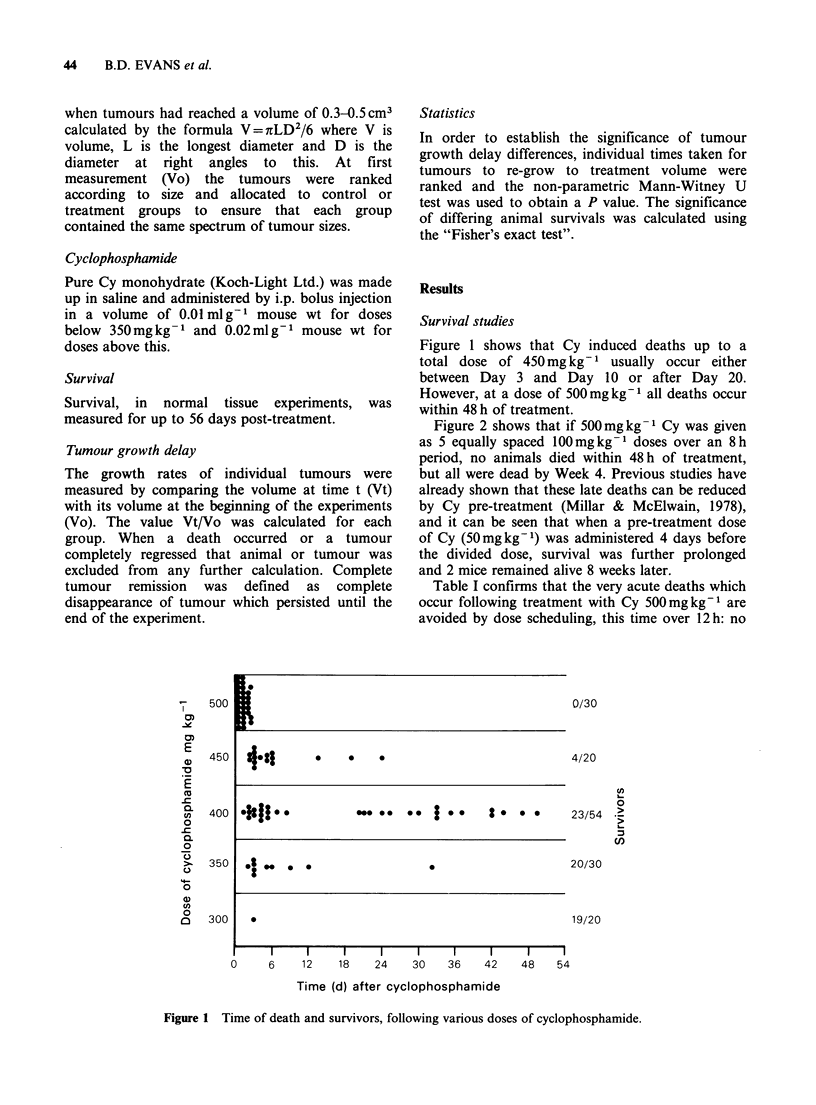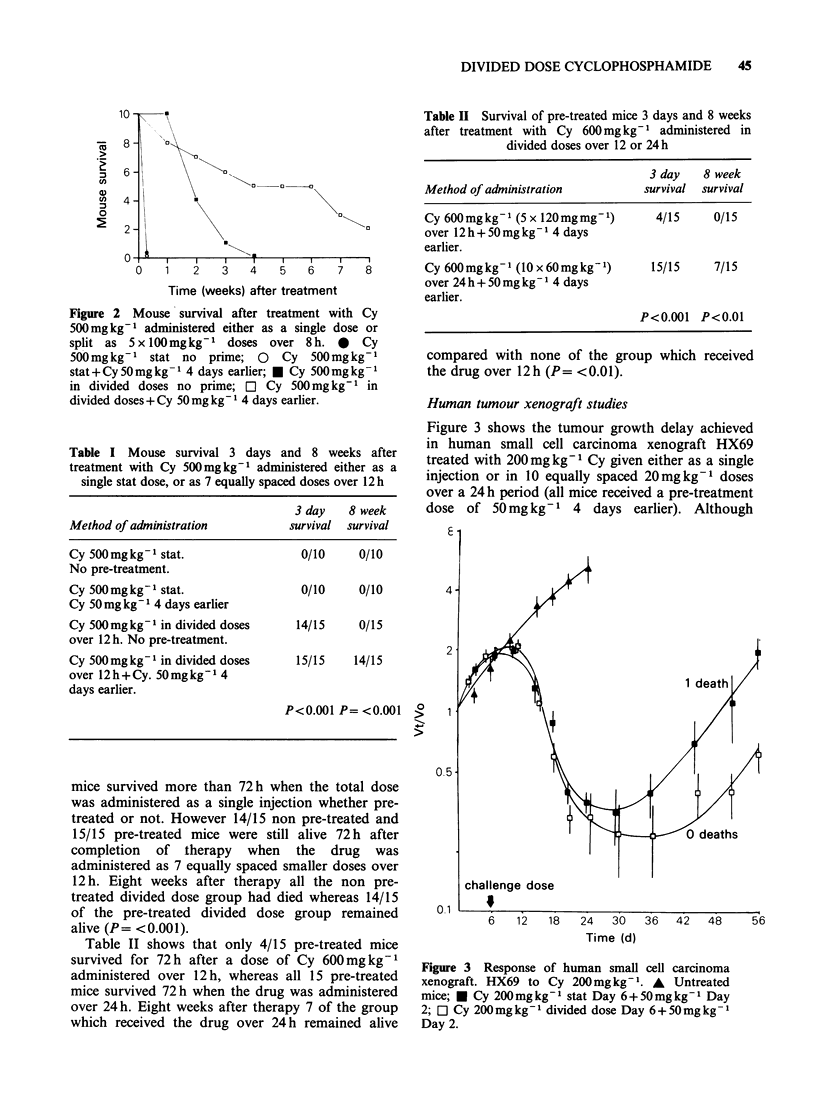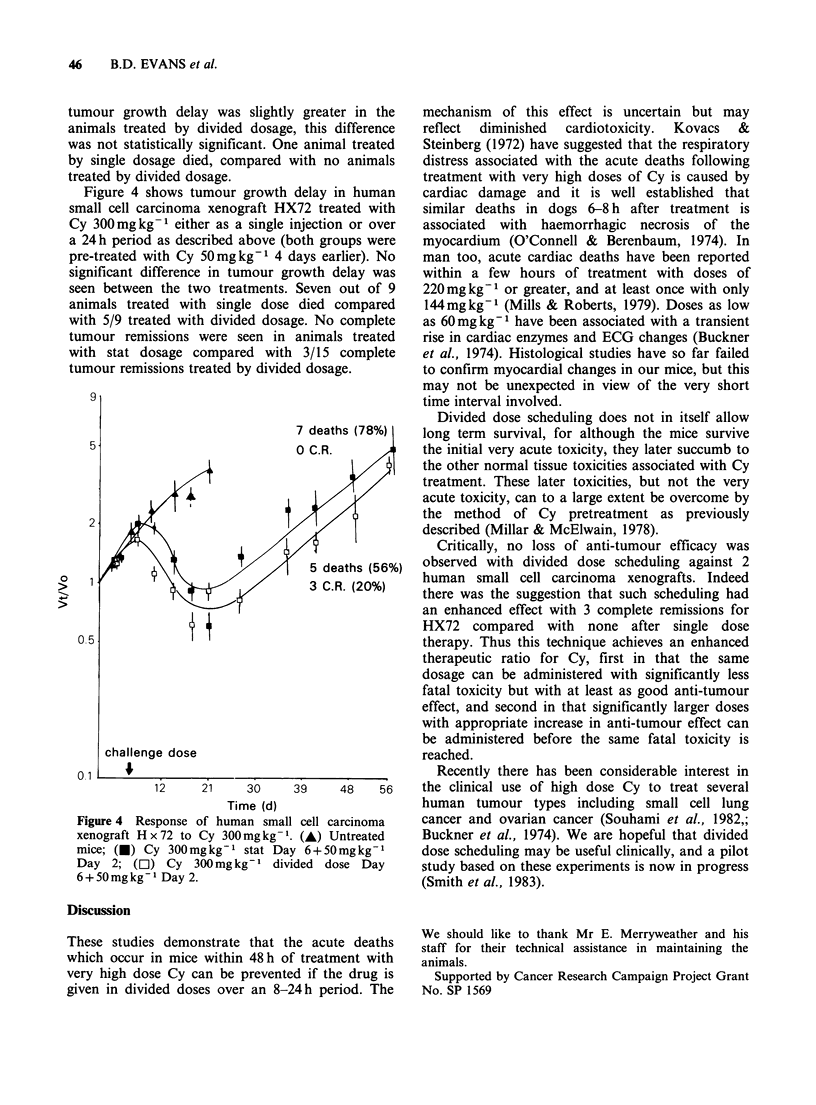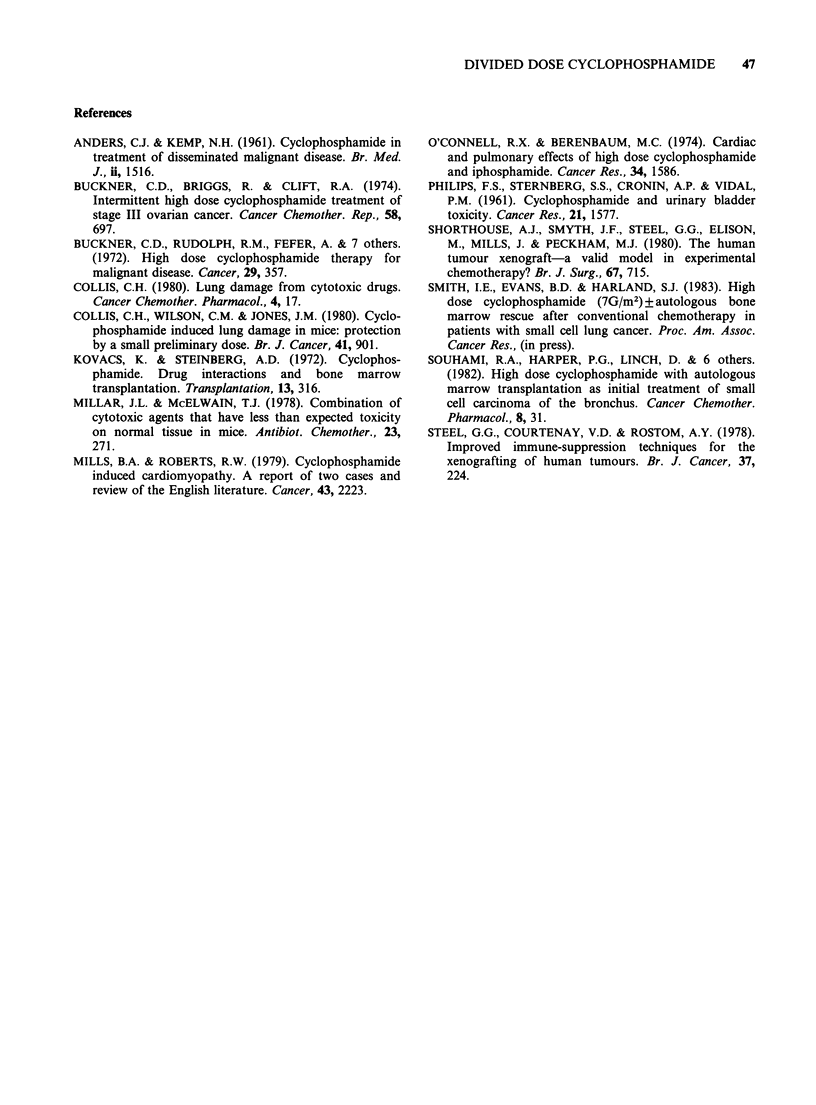# Prevention of acute deaths in mice after very high dose cyclophosphamide by divided dose schedule.

**DOI:** 10.1038/bjc.1984.7

**Published:** 1984-01

**Authors:** B. D. Evans, I. E. Smith, R. D. Clutterbuck, J. L. Millar

## Abstract

Very high dose cyclophosphamide (Cy) (500-600 mg kg-1) given by single bolus i.p. injection in mice caused acute deaths in all animals within 48 h of treatment (0/10 survivors). These acute deaths were abolished or very significantly reduced if Cy was administered in divided dosage over 8 h (10/10 survivors) or 12 h (14/15 survivors). The effect was maintained at doses of up to 600 mg kg-1 administered in divided dosage over 24 h (15/15 survivors). In 2 human small cell carcinoma xenografts anti-tumour efficacy was not diminished by divided dosage. In both xenografts tumour growth delay was enhanced, although not significantly so, when treated with divided dosage compared with single dose, and in one of the xenografts 3 complete remissions were achieved with divided dosage compared with none after single dosage. It is postulated that the underlying mechanism concerns diminished cardiotoxicity. These results may have significance in clinical studies investigating very high dose Cy.


*Br. J. Cancer* (1984), **49**, 43–47

### Prevention of acute deaths in mice after very high dose cyclophosphamide by divided dose schedule

B.D. Evans[1], I.E. Smith[1,2], R.D. Clutterbuck[2] & J.L. Millar[2]

[1]*Royal Marsden Hospital, Fulham Road, London, SW3,* [2]*Institute of Cancer Research, Downs Road, Sutton, Surrey.*

**Summary** Very high dose cyclophosphamide (Cy) (500–600 $mg\,kg^{-1}$) given by single bolus i.p. injection in mice caused acute deaths in all animals within 48 h of treatment (0/10 survivors). These acute deaths were abolished or very significantly reduced if Cy was administered in divided dosage over 8 h (10/10 survivors) or 12 h (14/15 survivors). The effect was maintained at doses of up to 600 $mg\,kg^{-1}$ administered in divided dosage over 24 h (15/15 survivors). In 2 human small cell carcinoma xenografts anti-tumour efficacy was not diminished by divided dosage. In both xenografts tumour growth delay was enhanced, although not significantly so, when treated with divided dosage compared with single dose, and in one of the xenografts 3 complete remissions were achieved with divided dosage compared with none after single dosage. It is postulated that the underlying mechanism concerns diminished cardiotoxicity. These results may have significance in clinical studies investigating very high dose Cy.

Cyclophosphamide (Cy) is toxic to several important normal tissues including bone marrow (Anders & Kemp, 1961), urothelium (Philips *et al.*, 1961), lung in mice (Collis *et al.*, 1980) and occasionally in man (Collis, 1980), and at very high doses, cardiac muscle (Mills & Roberts, 1979). This toxicity is dose related, and in mice the time of death after lethal dosage also depends on the dose administered. At doses of Cy of up to 450 $mg\,kg^{-1}$ our studies show that most deaths occur between days 3–10 post-treatment. This corresponds to the presence of Cy induced neutropenia and urothelial damage (personal observation). At this dosage however some later deaths are also seen from about day 20 onwards and this corresponds to the period of respiratory distress and histologically demonstrated lung damage (Collis *et al.*, 1980).

At doses of Cy $>450\,mg\,kg^{-1}$, mice die within a few hours of treatment, and it has been suggested that these deaths are associated with cardiac damage (Kovacs & Steinberg, 1972). Similar acute deaths have been observed in dogs and have been shown at necropsy to be associated with cardiac damage (O'Connell & Berenbaum, 1972). Acute cardiac deaths occurring within a few days of very high dose Cy treatment have also been reported in man (Mills & Roberts, 1979).

In this paper, we describe attempts to prevent these acute deaths associated with very high dose Cy by administering the drug in divided dosage. We also describe the effects of such treatment on two human small cell carcinoma xenografts grown in immune deprived mice.

Correspondence: B.D. Evans

Received 8 July 1983; accepted 19 September 1983.

### Materials and methods

### *Mice*

C57BL mice bred at the Institute of Cancer Research, Pollards Wood, were used for all normal tissue studies.

CBA/lac mice immune suppressed by thymectomy at 4 weeks of age followed 4 weeks later by 9 Gy whole body irradiation were used for human tumour xenograft studies. The mice were pre-treated with cytosine arabinoside 2 days before the total body irradiation in order to enhance bone marrow recovery, as previously described (Steel *et al.*, 1978).

### *Human tumour xenograft studies*

Two human small cell carcinoma xenografts HX69 and HX72 originally established from material obtained by surgical biopsy were used in these studies. The tumours were in early passage, from serial passages 6–14, and had been shown to retain their human histological and chromosomal characteristics, and to have therapeutic responses to cytotoxic drugs similar to that seen in the patients from which the original biopsies were obtained (Shorthouse *et al.*, 1980). At the start of each experiment tumour fragments measuring 2–3 mm. were bilaterally implanted s.c. into the flanks of 8–10 weeks old immune-suppressed CBA/lac mice. Tumour growth delay experiments were begun

© The Macmillan Press Ltd., 1984

when tumours had reached a volume of 0.3–0.5 $cm^3$ calculated by the formula $V = \pi LD^2/6$ where V is volume, L is the longest diameter and D is the diameter at right angles to this. At first measurement (Vo) the tumours were ranked according to size and allocated to control or treatment groups to ensure that each group contained the same spectrum of tumour sizes.

*Cyclophosphamide*

Pure Cy monohydrate (Koch-Light Ltd.) was made up in saline and administered by i.p. bolus injection in a volume of $0.01 \, ml \, g^{-1}$ mouse wt for doses below $350 \, mg \, kg^{-1}$ and $0.02 \, ml \, g^{-1}$ mouse wt for doses above this.

*Survival*

Survival, in normal tissue experiments, was measured for up to 56 days post-treatment.

*Tumour growth delay*

The growth rates of individual tumours were measured by comparing the volume at time t (Vt) with its volume at the beginning of the experiments (Vo). The value Vt/Vo was calculated for each group. When a death occurred or a tumour completely regressed that animal or tumour was excluded from any further calculation. Complete tumour remission was defined as complete disappearance of tumour which persisted until the end of the experiment.

*Statistics*

In order to establish the significance of tumour growth delay differences, individual times taken for tumours to re-grow to treatment volume were ranked and the non-parametric Mann-Witney U test was used to obtain a $P$ value. The significance of differing animal survivals was calculated using the "Fisher's exact test".

### Results

*Survival studies*

Figure 1 shows that Cy induced deaths up to a total dose of $450 \, mg \, kg^{-1}$ usually occur either between Day 3 and Day 10 or after Day 20. However, at a dose of $500 \, mg \, kg^{-1}$ all deaths occur within 48 h of treatment.

Figure 2 shows that if $500 \, mg \, kg^{-1}$ Cy was given as 5 equally spaced $100 \, mg \, kg^{-1}$ doses over an 8 h period, no animals died within 48 h of treatment, but all were dead by Week 4. Previous studies have already shown that these late deaths can be reduced by Cy pre-treatment (Millar & McElwain, 1978), and it can be seen that when a pre-treatment dose of Cy ($50 \, mg \, kg^{-1}$) was administered 4 days before the divided dose, survival was further prolonged and 2 mice remained alive 8 weeks later.

Table I confirms that the very acute deaths which occur following treatment with Cy $500 \, mg \, kg^{-1}$ are avoided by dose scheduling, this time over 12 h: no

**Figure 1** Time of death and survivors, following various doses of cyclophosphamide.

**Figure 2** Mouse survival after treatment with Cy $500 mg kg^{-1}$ administered either as a single dose or split as $5 \times 100 mg kg^{-1}$ doses over 8 h. ● Cy $500 mg kg^{-1}$ stat no prime; ○ Cy $500 mg kg^{-1}$ stat + Cy $50 mg kg^{-1}$ 4 days earlier; ■ Cy $500 mg kg^{-1}$ in divided doses no prime; □ Cy $500 mg kg^{-1}$ in divided doses + Cy $50 mg kg^{-1}$ 4 days earlier.

**Table I** Mouse survival 3 days and 8 weeks after treatment with Cy $500 mg kg^{-1}$ administered either as a single stat dose, or as 7 equally spaced doses over 12 h

| *Method of administration* | *3 day survival* | *8 week survival* |
|---|---|---|
| Cy $500 mg kg^{-1}$ stat. No pre-treatment. | 0/10 | 0/10 |
| Cy $500 mg kg^{-1}$ stat. Cy $50 mg kg^{-1}$ 4 days earlier | 0/10 | 0/10 |
| Cy $500 mg kg^{-1}$ in divided doses over 12 h. No pre-treatment. | 14/15 | 0/15 |
| Cy $500 mg kg^{-1}$ in divided doses over 12 h + Cy. $50 mg kg^{-1}$ 4 days earlier. | 15/15 | 14/15 |
| | $P<0.001$ | $P=<0.001$ |

mice survived more than 72 h when the total dose was administered as a single injection whether pre-treated or not. However 14/15 non pre-treated and 15/15 pre-treated mice were still alive 72 h after completion of therapy when the drug was administered as 7 equally spaced smaller doses over 12 h. Eight weeks after therapy all the non pre-treated divided dose group had died whereas 14/15 of the pre-treated divided dose group remained alive ($P=<0.001$).

Table II shows that only 4/15 pre-treated mice survived for 72 h after a dose of Cy $600 mg kg^{-1}$ administered over 12 h, whereas all 15 pre-treated mice survived 72 h when the drug was administered over 24 h. Eight weeks after therapy 7 of the group which received the drug over 24 h remained alive

**Table II** Survival of pre-treated mice 3 days and 8 weeks after treatment with Cy $600 mg kg^{-1}$ administered in divided doses over 12 or 24 h

| *Method of administration* | *3 day survival* | *8 week survival* |
|---|---|---|
| Cy $600 mg kg^{-1}$ ($5 \times 120 mg mg^{-1}$) over 12 h + $50 mg kg^{-1}$ 4 days earlier. | 4/15 | 0/15 |
| Cy $600 mg kg^{-1}$ ($10 \times 60 mg kg^{-1}$) over 24 h + $50 mg kg^{-1}$ 4 days earlier. | 15/15 | 7/15 |
| | $P<0.001$ | $P<0.01$ |

compared with none of the group which received the drug over 12 h ($P=<0.01$).

*Human tumour xenograft studies*

Figure 3 shows the tumour growth delay achieved in human small cell carcinoma xenograft HX69 treated with $200 mg kg^{-1}$ Cy given either as a single injection or in 10 equally spaced $20 mg kg^{-1}$ doses over a 24 h period (all mice received a pre-treatment dose of $50 mg kg^{-1}$ 4 days earlier). Although

**Figure 3** Response of human small cell carcinoma xenograft. HX69 to Cy $200 mg kg^{-1}$. ▲ Untreated mice; ■ Cy $200 mg kg^{-1}$ stat Day 6 + $50 mg kg^{-1}$ Day 2; □ Cy $200 mg kg^{-1}$ divided dose Day 6 + $50 mg kg^{-1}$ Day 2.

tumour growth delay was slightly greater in the animals treated by divided dosage, this difference was not statistically significant. One animal treated by single dosage died, compared with no animals treated by divided dosage.

Figure 4 shows tumour growth delay in human small cell carcinoma xenograft HX72 treated with Cy 300 mg $kg^{-1}$ either as a single injection or over a 24 h period as described above (both groups were pre-treated with Cy 50 mg $kg^{-1}$ 4 days earlier). No significant difference in tumour growth delay was seen between the two treatments. Seven out of 9 animals treated with single dose died compared with 5/9 treated with divided dosage. No complete tumour remissions were seen in animals treated with stat dosage compared with 3/15 complete tumour remissions treated by divided dosage.

**Figure 4** Response of human small cell carcinoma xenograft H x 72 to Cy 300 mg $kg^{-1}$. (▲) Untreated mice; (■) Cy 300 mg $kg^{-1}$ stat Day 6 + 50 mg $kg^{-1}$ Day 2; (□) Cy 300 mg $kg^{-1}$ divided dose Day 6 + 50 mg $kg^{-1}$ Day 2.

### Discussion

These studies demonstrate that the acute deaths which occur in mice within 48 h of treatment with very high dose Cy can be prevented if the drug is given in divided doses over an 8–24 h period. The mechanism of this effect is uncertain but may reflect diminished cardiotoxicity. Kovacs & Steinberg (1972) have suggested that the respiratory distress associated with the acute deaths following treatment with very high doses of Cy is caused by cardiac damage and it is well established that similar deaths in dogs 6–8 h after treatment is associated with haemorrhagic necrosis of the myocardium (O'Connell & Berenbaum, 1974). In man too, acute cardiac deaths have been reported within a few hours of treatment with doses of 220 mg $kg^{-1}$ or greater, and at least once with only 144 mg $kg^{-1}$ (Mills & Roberts, 1979). Doses as low as 60 mg $kg^{-1}$ have been associated with a transient rise in cardiac enzymes and ECG changes (Buckner *et al.*, 1974). Histological studies have so far failed to confirm myocardial changes in our mice, but this may not be unexpected in view of the very short time interval involved.

Divided dose scheduling does not in itself allow long term survival, for although the mice survive the initial very acute toxicity, they later succumb to the other normal tissue toxicities associated with Cy treatment. These later toxicities, but not the very acute toxicity, can to a large extent be overcome by the method of Cy pretreatment as previously described (Millar & McElwain, 1978).

Critically, no loss of anti-tumour efficacy was observed with divided dose scheduling against 2 human small cell carcinoma xenografts. Indeed there was the suggestion that such scheduling had an enhanced effect with 3 complete remissions for HX72 compared with none after single dose therapy. Thus this technique achieves an enhanced therapeutic ratio for Cy, first in that the same dosage can be administered with significantly less fatal toxicity but with at least as good anti-tumour effect, and second in that significantly larger doses with appropriate increase in anti-tumour effect can be administered before the same fatal toxicity is reached.

Recently there has been considerable interest in the clinical use of high dose Cy to treat several human tumour types including small cell lung cancer and ovarian cancer (Souhami *et al.*, 1982,; Buckner *et al.*, 1974). We are hopeful that divided dose scheduling may be useful clinically, and a pilot study based on these experiments is now in progress (Smith *et al.*, 1983).

We should like to thank Mr E. Merryweather and his staff for their technical assistance in maintaining the animals.

Supported by Cancer Research Campaign Project Grant No. SP 1569

### References

ANDERS, C.J. & KEMP, N.H. (1961). Cyclophosphamide in treatment of disseminated malignant disease. *Br. Med. J.*, **ii**, 1516.

BUCKNER, C.D., BRIGGS, R. & CLIFT, R.A. (1974). Intermittent high dose cyclophosphamide treatment of stage III ovarian cancer. *Cancer Chemother. Rep.*, **58**, 697.

BUCKNER, C.D., RUDOLPH, R.M., FEFER, A. & 7 others. (1972). High dose cyclophosphamide therapy for malignant disease. *Cancer*, **29**, 357.

COLLIS, C.H. (1980). Lung damage from cytotoxic drugs. *Cancer Chemother. Pharmacol.*, **4**, 17.

COLLIS, C.H., WILSON, C.M. & JONES, J.M. (1980). Cyclophosphamide induced lung damage in mice: protection by a small preliminary dose. *Br. J. Cancer*, **41**, 901.

KOVACS, K. & STEINBERG, A.D. (1972). Cyclophosphamide. Drug interactions and bone marrow transplantation. *Transplantation*, **13**, 316.

MILLAR, J.L. & MCELWAIN, T.J. (1978). Combination of cytotoxic agents that have less than expected toxicity on normal tissue in mice. *Antibiot. Chemother.*, **23**, 271.

MILLS, B.A. & ROBERTS, R.W. (1979). Cyclophosphamide induced cardiomyopathy. A report of two cases and review of the English literature. *Cancer*, **43**, 2223.

O'CONNELL, R.X. & BERENBAUM, M.C. (1974). Cardiac and pulmonary effects of high dose cyclophosphamide and iphosphamide. *Cancer Res.*, **34**, 1586.

PHILIPS, F.S., STERNBERG, S.S., CRONIN, A.P. & VIDAL, P.M. (1961). Cyclophosphamide and urinary bladder toxicity. *Cancer Res.*, **21**, 1577.

SHORTHOUSE, A.J., SMYTH, J.F., STEEL, G.G., ELISON, M., MILLS, J. & PECKHAM, M.J. (1980). The human tumour xenograft—a valid model in experimental chemotherapy? *Br. J. Surg.*, **67**, 715.

SMITH, I.E., EVANS, B.D. & HARLAND, S.J. (1983). High dose cyclophosphamide ($7G/m^2$) $\pm$ autologous bone marrow rescue after conventional chemotherapy in patients with small cell lung cancer. *Proc. Am. Assoc. Cancer Res.*, (in press).

SOUHAMI, R.A., HARPER, P.G., LINCH, D. & 6 others. (1982). High dose cyclophosphamide with autologous marrow transplantation as initial treatment of small cell carcinoma of the bronchus. *Cancer Chemother. Pharmacol.*, **8**, 31.

STEEL, G.G., COURTENAY, V.D. & ROSTOM, A.Y. (1978). Improved immune-suppression techniques for the xenografting of human tumours. *Br. J. Cancer*, **37**, 224.